# Supplementary material for: Expanding the Therapeutic Window of EGFR-Targeted PE24 Immunotoxin for EGFR-Overexpressing Cancers by Tailoring the EGFR Binding Affinity
Source: Int J Mol Sci. 2022 Dec 13;23(24):15820. doi: 10.3390/ijms232415820 (PMC9779439; doi:10.3390/ijms232415820)
Supplement: Supplementary file 1 [file ijms-23-15820-s001.zip › ijms-2025224-supplementary.pdf]

Supplementary Material

Expanding the therapeutic window of EGFR-targeted PE24 immunotoxin for EGFR-overexpressing cancers by tailoring the EGFR binding affinity

Sei-Yong Jun, Dae-Seong Kim, and Yong-Sung Kim

Inventory of Supplementary Information

Supplementary Figure 1

Supplementary Figure 2

Supplementary Figure 3

Supplementary Figure 1

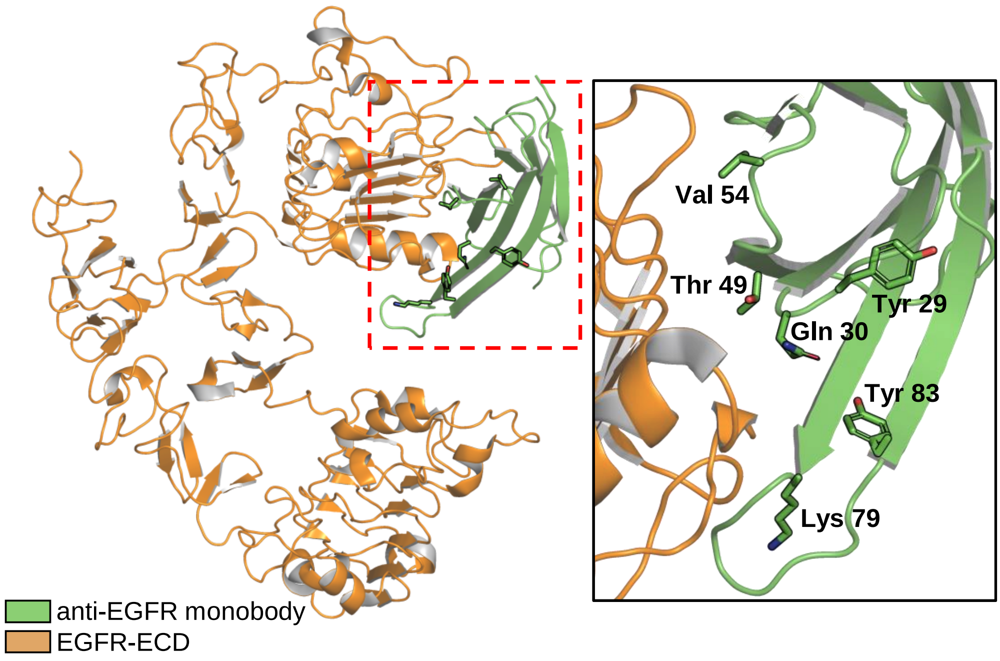

Supplementary Figure 1. The complex structure of EGFR-ECD (orange) and EGFR-targeting monobody (green) (PDB code 3QWQ) highlights the mutated residues used to construct the different monobody variants with varying affinities for EGFR. The images are illustrated using PyMOL (Schrödinger, Inc., NY, USA).

Supplementary Figure 2

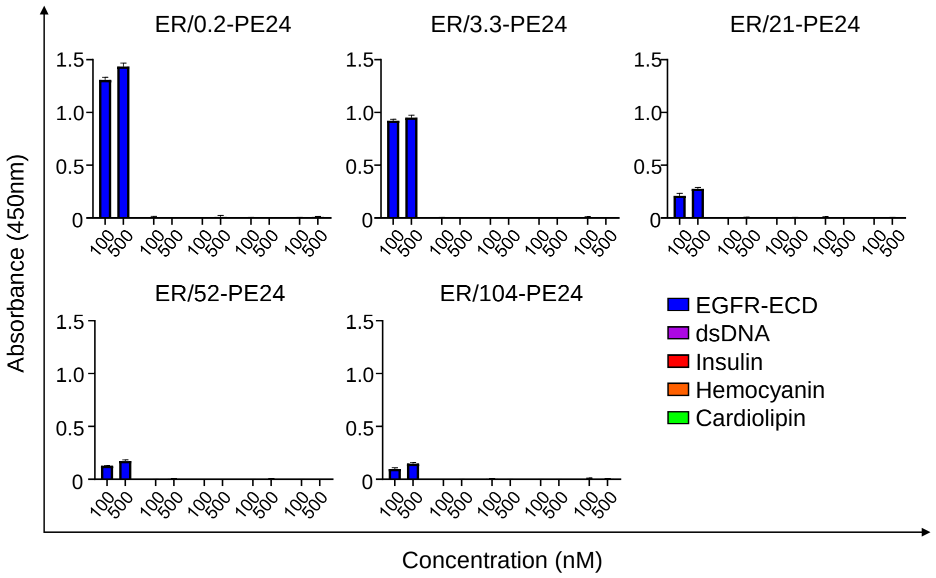

Supplementary Figure 2. Evaluation of nonspecific binding of the indicated ER-PE24 ITs to four off-target antigens (EGFR-ECD, double-stranded DNA (dsDNA), insulin, hemocyanin, and cardiolipin), as determined by ELISA. EGFR-ECD was used as a positive control. Error bars represent the mean ± SD.

Supplementary Figure 3

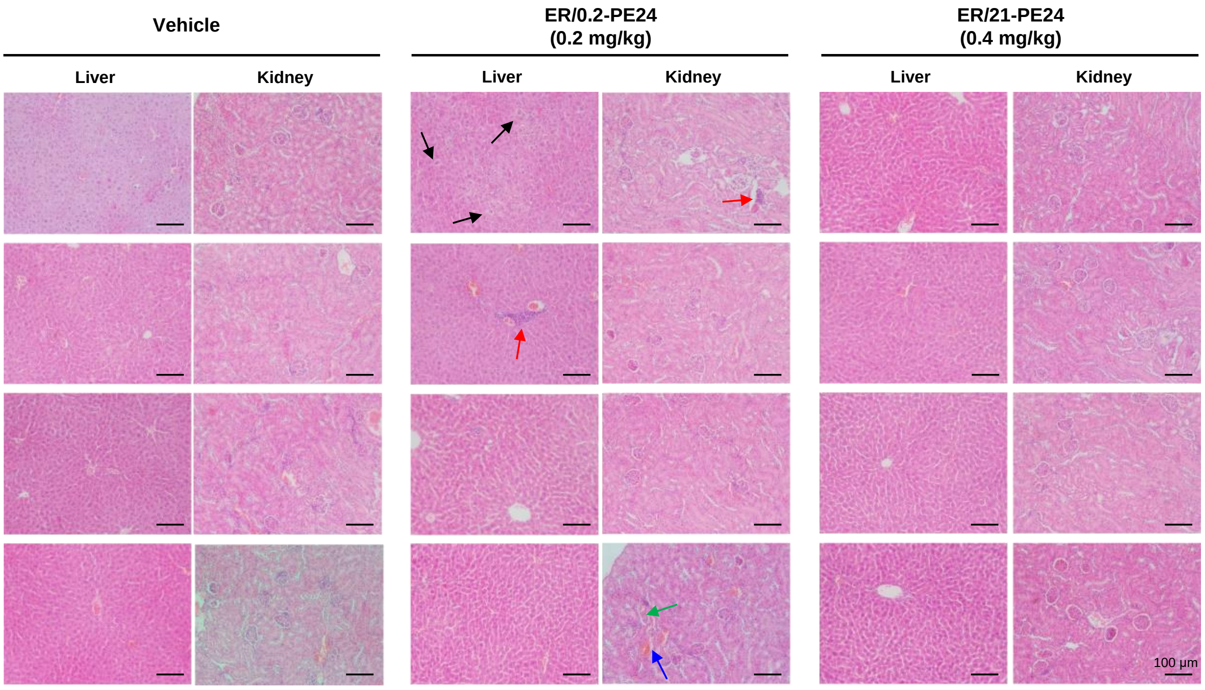

Supplementary Figure 3. Hematoxylin and eosin (H&E) staining of representative tissue sections from the liver and kidney of each group of TBM, performed at the end of treatment of either i.v. injection of vehicle (left), ER/0.2-PE24 at a dose of 0.2 mg/kg (middle), and ER/21-PE24 at a dose of 0.4 mg/kg (right) every other day. The abnormal histological features suggestive of hepatic toxicity or renal toxicity are indicated using arrows of different colors [Hepatocyte ballooning (black), inflammatory cell infiltration (red), tubular necrosis (Green), interstitial hemorrhage (blue)]. Magnification, ×200; scale bar, 100 μm.
